# Lack of Routine Health Care Among Adult Survivors of Childhood, Adolescent, and Young Adult Cancers (CAYA): A Nationally Representative Study of 4300 Survivors

**DOI:** 10.1002/cam4.70924

**Published:** 2025-05-07

**Authors:** Andrea C. Betts, Rebecca Eary, Bhaskar Thakur, Amy Hughes, Quiera S. Booker, L. Aubree Shay, Simon Craddock Lee, Daniel C. Bowers, Bijal A. Balasubramanian

**Affiliations:** ^1^ Department of Health Promotion and Behavioral Sciences, School of Public Health The University of Texas Health Science Center at Houston Dallas Texas USA; ^2^ Department of Family and Community Medicine University of Texas Southwestern Medical Center Dallas Texas USA; ^3^ Peter O'Donnell Jr. School of Public Health University of Texas Southwestern Medical Center Dallas Texas USA; ^4^ Division of Cancer Epidemiology and Genetics National Cancer Institute Bethesda Maryland USA; ^5^ Department of Epidemiology, School of Public Health The University of Texas Health Science Center at Houston Dallas Texas USA; ^6^ Department of Population Health University of Kansas Cancer Center and School of Medicine Kansas City Kansas USA; ^7^ Department of Pediatrics University of Texas Southwestern Medical Center Dallas Texas USA; ^8^ Department of Pediatric Hematology/Oncology Children's Health Dallas Texas USA; ^9^ Institute for Implementation Science Dallas Texas USA

**Keywords:** adolescent young adult cancer, cancer survivorship, childhood cancer, health care transitions, health services research, primary care

## Abstract

**Background:**

Survivors of childhood, adolescent, and young adult cancers (CAYA) experience lifelong health risks and accelerated aging. We examined routine health care (no routine checkup in the past year) among a nationally representative sample of CAYA survivors at different life stages.

**Methods:**

We pooled data from the Behavioral Risk Factor Surveillance System (BRFSS; 2012, 2014, 2016–2019). CAYA survivors (ages 0–39 at cancer diagnosis) were young adults (18–39 years), middle‐aged (40–64 years), or older adults (≥ 65 years) at the time of the survey. We estimated the prevalence of: (1) not receiving routine health care and (2) not having a personal doctor, overall and by age at survey. We used multivariable Poisson regression to identify factors associated with these outcomes.

**Results:**

We identified 4284 CAYA survivors: 884 young adults, 2201 middle‐aged, and 1199 older adults. More young adults were uninsured and unable to afford care, compared to other age groups. A higher proportion of young adults did not receive routine health care (35.9%, 95% CI 30.3–41.9) or have a personal doctor (25.6%, 95% CI 20.5–31.4), compared to middle‐aged or older CAYA survivors (*p* < 0.01). In multivariable models, being a young adult was strongly associated with (1) not receiving routine health care (aPR 1.82, 95% CI 1.24–2.67) and (2) not having a personal doctor (aPR 3.14, 95% CI 1.84–5.35).

**Conclusions:**

Younger CAYA survivors experience a triple threat of chronic conditions, modifiable risks, and disconnection from routine health care.

**Impact:**

Early interventions to facilitate care transitions are needed.

## Graphical abstract

We examined routine health care (no routine checkup in the past year) among a nationally representative sample of childhood, adolescent, and young adult (CAYA) cancer survivors at different life stages. A higher proportion of young adults did not receive routine health care or have a personal doctor, compared to middle‐aged or older CAYA survivors. In multivariable models, being a young adult was strongly associated with (1) not receiving routine health care and (2) not having a personal doctor.

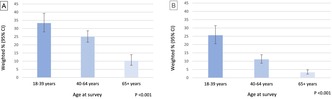

## 1 Introduction

In the United States, over two million survivors [1, 2] of childhood, adolescent, and young adult cancers (CAYA survivors)—individuals diagnosed with cancer between birth and age 39 years [1]—experience the risk of late and long‐term effects of cancer and its treatment. These effects include the development of severe or life‐threatening chronic conditions and second cancers [3, 4, 5, 6, 7]. For example, exposure to chemotherapy and/or radiation increases risk of second cancers [5, 8, 9, 10] as well as heart and lung disease, neuropathy, infertility, kidney failure, neurocognitive effects, and osteopenia/osteoporosis, all of which may require early intervention and specialist care [11, 12]. These effects carry significant physical, financial, and mental health ramifications [13, 14] and may be further compounded by the emergence of aging‐associated diseases [4, 15].

High quality survivorship care is needed to monitor and address these risks and reduce the burden of morbidity and mortality in CAYA survivors. However, despite nearly 20 years of national emphasis on developing survivorship care practices [16, 17], many CAYA survivors do not receive routine health care after completion of cancer treatment [18]. Lack of follow‐up care has been associated with: lack of insurance, inability to afford care, lower income, and living in lower socioeconomic status communities [19, 20, 21]. Although there is considerable literature that focuses on access to care among survivors of childhood cancers, comparatively few studies have included survivors of young adult cancers [22, 23, 24, 25, 26], who are historically understudied. However, the incidence of cancers in this age group is increasing [27].

Further, patterns of routine health care among CAYA survivors across the lifespan are not well understood. Given that a cancer diagnosis both elevates health risks across the lifespan and disrupts developmental norms for CAYA survivors, there is a need to understand how these survivors engage in care at different life stages, and when they may be most at risk of disconnection from care. Such knowledge may inform future programs to facilitate retention within the health care system. The purpose of this analysis is to characterize routine health care at different life stages using a large, nationally representative sample of CAYA survivors. Specifically, we used data from the U.S. Centers for Disease Control and Prevention's (CDC's) Behavioral Risk Factor Surveillance System (BRFSS) to estimate the prevalence of and factors associated with: (1) not receiving routine health care, and (2) not having a personal doctor, among survivors of CAYA cancers who were young adults (18–39 years), middle‐aged (40–64 years), and older adults (≥ 65 years) at the time of the survey. In addition, we estimate the prevalence of chronic conditions and modifiable risk factors across the lifespan, to contextualize routine care needs and inform care planning.

## 2 Materials and Methods

### 2.1 Data Source

This cross‐sectional study utilized pooled data from the BRFSS [28], an annually administered, nationally representative health‐related telephone survey involving over 400,000 adult‐aged (≥ 18 years) U.S. residents. To address the complex survey design and facilitate nationally representative estimates, BRFSS employs weighting and iterative proportional fitting. In addition to the base questions in the survey, BRFSS fielded questions about cancer history and care in the cancer survivorship module in 2012, 2014, and 2016–2021.

### 2.2 Study Population

We pooled data from the BFRSS survey years 2012, 2014, and 2016–2019. We did not include survey years 2020 and 2021 because a considerable proportion of observations in the optional Cancer Survivorship module within our sample were missing during these pandemic years. We identified survivors of CAYA cancers as those reporting a cancer diagnosis between 0 and 39 years old. Similar to other studies [29, 30], we excluded those who reported a history of non‐melanoma skin cancer as their only diagnosed cancer (*n* = 1127). We stratified CAYA survivors into one of three categories based on their age at the time of survey completion: young adults (18–39 years), middle age (40–64 years), and older adults (≥ 65 years). These groups were defined based on the age range commonly used to define young adults with cancer (18–39 years) [31] and the age of Medicare eligibility (≥ 65 years).

### 2.3 Routine Health Care (Main Dependent Variable)

We examined routine health care among CAYA survivors using items from both the core and the optional Cancer Survivorship module of the BRFSS survey. Routine health care was assessed based on responses to the question: *How long has it been since you last visited a doctor for a routine checkup? A routine checkup is a general physical exam, not an exam for a specific injury, illness, or condition*. Response options included: within the past year (anytime less than 12 months ago); within the past 2 years (> 1 year but less than 2 years ago); within the past 5 years (> 2 years but less than 5 years ago); 5 or more years ago; don't know/not sure; never; and refused. Having a personal doctor was assessed based on responses to the question: *Do you have one person you think of as your personal doctor? Or is there more than one, or no person as your personal doctor/healthcare provider?* Response options included: yes, only one; more than one; no; don't know/not sure; and refused.

### 2.4 Sociodemographic and Clinical Characteristics and Modifiable Risk Factors (Independent Variables)

We measured sociodemographic and clinical characteristics and modifiable risk factors using items from both the core and optional survivorship modules. Sociodemographic characteristics included age at first diagnosis of cancer, sex at birth, race and ethnicity, insurance coverage, marital status, educational level, annual household income, inability to afford needed care, and receipt of survivorship care plan. Primary cancer diagnosis is reported for those with a history of only one cancer because BRFSS asks only about the most recent type of cancer. Clinical characteristics included the history of any reported chronic condition (yes/no), the number of reported chronic conditions, and the number of reported cancers. Modifiable risk factors included body mass index (BMI) and obesity, smoking status, and alcohol use. Alcohol use was defined by the CDC for the BRFSS survey and included the following: “any use,” “heavy drinking” (> 14 drinks/week for males; > 7 drinks/week for females), and “binge drinking” (≥ 5 drinks for males on one occasion; ≥ 4 drinks for females on one occasion) as defined for men and women based on Centers for Disease Control guidelines [32].

### 2.5 Statistical Analysis

We estimated weighted prevalence of sociodemographic and clinical characteristics, modifiable risk factors, and use of routine health care among survivors of CAYA cancers, overall and stratified by age at survey (18–39 years, 40–64 years, ≥ 65 years). We used survey‐based multivariable Poisson regression to identify sociodemographic, clinical, and modifiable risk factors associated with (1) lack of routine health care, defined as not having a routine checkup in the past year, and (2) not having a personal doctor. Adjusted prevalence ratios (aPRs) and 95% confidence intervals were estimated using this method, with models limited to observations without missing data on the primary outcomes. (Missingness was 1.7% for routine care and < 0.01% for personal doctor.) For regression analyses, we dichotomized response options for both dependent variables. Lack of routine care was defined as > 1 year since the last routine checkup. Not having a personal doctor was defined as a “no” response to that question. Independent variables included age at cancer diagnosis, age at survey administration, sex at birth, race/ethnicity, marital status, any health insurance, annual income, smoking status, alcohol use in the past 30 days (yes/no), obesity (yes/no based on BMI ≥ 30), number of comorbidities, number of cancers, and receipt of survivorship care plan (yes/no/missing). Variables were selected based on review of existing literature. In addition, to assess the association of cancer type with routine care outcomes, we similarly fit multivariable Poisson regression models among the subset of CAYA survivors whose primary cancer diagnosis is known. Statistical analyses were conducted using STATA version 18 [33]. This study received an exemption from the institutional review board at the University of Texas Health Science Center at Houston (HSC‐SPH‐24‐0459).

## 3 Results

### 3.1 Sociodemographics, Clinical Characteristics, and Modifiable Risk Factors

We identified 4284 CAYA survivors, among whom 884 were young adults at the time of the survey, 2201 were middle age, and 1199 were older adults (Table 1). The median age at first diagnosis of cancer was 29 years (IQR 22–34), and a majority of the sample were female at birth (75.2%). The distribution of race and ethnicity varied by age at survey, with somewhat greater diversity among young adults. Cancer types were diverse (Table S1A), with cervical cancer, melanoma, and breast cancer being the most common. Young adult CAYA survivors were less likely than older adult CAYA survivors to be married or to have any insurance (both *p* < 0.01). Most survivors had an annual income < $50,000, and 38.8% of the young adult group had an income < $25,000. One in four young adult CAYAs (28.1%, 95% CI 23.2, 33.8) and middle age CAYAs (25.1%, 95% CI 22.4, 29.4) were unable to afford needed care. The prevalence of chronic conditions and modifiable risk factors was high overall and increased with age group (Table 2). Notably, 43.2% of survivors who were young adults at the time of the survey had a chronic condition. Most survivors were either overweight (28.6%) or obese (34.2%), and many were current smokers (40.2% of young adults and 31.3% of middle age CAYA survivors). Most young adult CAYA survivors drank alcohol in the past 30 days (54.1%), and nearly 1 in 5 reported binge drinking; the prevalence of these behaviors was lower among middle age and older survivors.

**TABLE 1:** Sociodemographic and cancer characteristics of childhood, adolescent, and young adult (CAYA) cancer survivors, by age at survey (*n* = 4284).

	Age at survey				
	18–39 years (*n* = 884)	40–64 years (*n* = 2201)	65+ years (*n* = 1199)	Total	*p*
Age at diagnosis					< 0.01
0–14 years	95 (12.2%)	59 (2.3%)	48 (4.4%)	202 (5.8%)	
15–19 years	170 (19.6%)	152 (6.9%)	44 (2.7%)	366 (10.3%)	
20–24 years	174 (21.9%)	332 (15.5%)	167 (11.9%)	673 (17.0%)	
25–29 years	210 (21.0%)	440 (17.5%)	210 (14.9%)	860 (18.2%)	
30–34 years	158 (18.0%)	535 (27.2%)	350 (31.7%)	1043 (25.0%)	
35–39 years	77 (7.3%)	683 (30.5%)	380 (34.5%)	1140 (23.7%)	
Sex at birth					
Male	191 (25.4%)	445 (25.1%)	217 (22.0%)	853 (24.7%)	0.74
Female	693 (74.6%)	1754 (74.8%)	982 (78.0%)	3429 (75.2%)	
Missing	0 (0.0%)	2 (0.1%)	0 (0.0%)	2 (0.1%)	
Race and ethnicity					< 0.01
American Indian/Alaska Native	35 (2.0%)	77 (1.6%)	37 (1.1%)	149 (1.7%)	
Asian/Native Hawaiian/Pacific Islander	11 (2.4%)	13 (0.3%)	8 (0.7%)	32 (1.0%)	
Hispanic (any race)	45 (5.5%)	57 (5.6%)	8 (0.2%)	110 (4.7%)	
Multiracial/unspecified Non‐Hispanic	53 (3.9%)	83 (2.1%)	33 (1.2%)	169 (2.6%)	
Non‐Hispanic Black	53 (9.3%)	102 (6.0%)	50 (5.7%)	205 (7.0%)	
Non‐Hispanic White	675 (75.1%)	1843 (82.8%)	1044 (88.1%)	3562 (81.1%)	
Not sure/missing	12 (1.7%)	26 (1.7%)	19 (3.0%)	57 (1.9%)	
Education					
High school or less	313 (43.3%)	788 (43.9%)	492 (48.3%)	1593 (44.4%)	0.80
Attended college	283 (33.1%)	691 (32.8%)	367 (31.5%)	1341 (32.7%)	
Graduated college	288 (23.5%)	719 (23.2%)	340 (20.2%)	1347 (22.9%)	
Missing	0 (0.0%)	3 (0.1%)	0 (0.0%)	3 (0.1%)	
Marital status					
Married	410 (40.7%)	1211 (56.7%)	491 (49.5%)	2112 (50.5%)	< 0.01
Divorced/separated/widowed	180 (17.6%)	751 (30.2%)	649 (45.2%)	1580 (28.4%)	
Never married/unmarried couple	293 (41.6%)	230 (12.7%)	54 (5.1%)	577 (20.8%)	
Missing	1 (0.1%)	9 (0.4%)	5 (0.3%)	15 (0.3%)	
Any insurance at survey					
Yes	746 (81.5%)	1945 (88.1%)	1178 (98.5%)	3869 (87.6%)	< 0.01
No	135 (18.3%)	254 (11.8%)	21 (1.5%)	410 (12.3%)	
Not sure/missing	3 (0.2%)	2 (0.0%)	0 (0.0%)	5 (0.1%)	
Annual income					
< $25,000	313 (38.8%)	687 (31.5%)	414 (29.0%)	1414 (33.5%)	< 0.01
$25,000–< $50,000	190 (21.7%)	406 (18.5%)	294 (25.9%)	890 (20.6%)	
≥ $50,000	294 (28.8%)	898 (41.9%)	282 (26.6%)	1474 (35.4%)	
Not sure/missing	87 (10.7%)	210 (8.1%)	209 (18.4%)	506 (10.5%)	
Annual income (among known)					
< $25,000	313 (43.5%)	687 (34.3%)	414 (35.6%)	1414 (37.4%)	< 0.01
$25,000–< $50,000	190 (24.3%)	406 (20.1%)	294 (31.8%)	890 (23.1%)	
≥ $50,000	294 (32.3%)	898 (45.6%)	282 (32.7%)	1474 (39.5%)	
Inability to afford care^a^					< 0.01
Yes	258 (28.2%)	476 (25.8%)	87 (7.2%)	821 (23.7%)	
No	626 (71.8%)	1722 (74.2%)	1108 (92.6%)	3456 (76.2%)	
Don't know/not sure/missing	0 (0.0%)	3 (0.1%)	4 (0.2%)	7 (0.1%)	
Survivorship care plan^b^					< 0.01
Yes	230 (26.3%)	491 (19.6%)	175 (15.6%)	896 (21.1%)	
No	333 (36.2%)	1026 (45.8%)	611 (50.3%)	1970 (43.4%)	
Missing	321 (37.4%)	684 (34.6%)	413 (34.1%)	1418 (35.4%)	

^a^ Inability to afford care was based on a “yes” response to the question: “Was there a time in the past 12 months when you needed to see a doctor but could not because of cost?”.

^b^ Similar to other studies [34], survivorship care plan was based on yes to both of the following questions: “Did any doctor, nurse, or other health professional ever give you a written summary of all the cancer treatments that you received?” and “Have you ever received instructions from a doctor, nurse, or other health professional about where you should return or who you should see for routine cancer checkups after completing your treatment for cancer?”.

**TABLE 2:** Clinical characteristics and modifiable risk factors of CAYA cancer survivors, by age at survey (*n* = 4284).

	Age at survey				
	18–39 years (*n* = 884)	40–64 years (*n* = 2201)	65+ years (*n* = 1199)	Total	*p*
	*n* (wt %)	*n* (wt %)	*n* (wt %)	*n* (wt %)	
Clinical characteristics					
Any chronic condition^a^	353 (43.2%)	1053 (46.6%)	687 (58.0%)	2093 (47.3%)	< 0.01
Number of chronic conditions^a^					< 0.01
0	531 (56.8%)	1148 (53.4%)	512 (42.0%)	2191 (52.7%)	
1	236 (28.9%)	560 (25.6%)	338 (29.7%)	1134 (27.3%)	
≥ 2	117 (14.3%)	493 (21.0%)	349 (28.2%)	959 (20.0%)	
Number of cancers					< 0.01
1	797 (91.2%)	1732 (78.6%)	766 (64.1%)	3295 (80.4%)	
2	73 (6.9%)	359 (16.7%)	328 (27.6%)	760 (15.2%)	
≥ 3	14 (1.9%)	110 (4.7%)	105 (8.4%)	229 (4.4%)	
Modifiable risk factors					
BMI, mean (SD)	27.7 (7.2)	29.3 (7.6)	28.4 (6.2)	28.7 (7.3)	< 0.01
BMI category					
Underweight	25 (3.9%)	46 (1.9%)	27 (2.4%)	98 (2.6%)	< 0.01
Normal weight	295 (35.0%)	625 (28.4%)	329 (24.2%)	1249 (29.9%)	
Overweight	246 (25.5%)	651 (27.7%)	393 (38.4%)	1290 (28.6%)	
Obese	263 (29.4%)	780 (37.9%)	392 (31.1%)	1435 (34.2%)	
Missing	55 (6.1%)	99 (4.1%)	58 (3.9%)	212 (4.7%)	
Smoking status					< 0.01
Current	320 (40.2%)	656 (31.3%)	171 (14.7%)	1147 (31.6%)	
Former	174 (16.5%)	604 (28.4%)	489 (40.5%)	1267 (26.5%)	
Never	388 (43.2%)	938 (40.2%)	531 (44.3%)	1857 (41.8%)	
Alcohol use					
Any alcohol in past 30 days	486 (54.1%)	1064 (48.9%)	414 (34.9%)	1964 (48.4%)	< 0.01
Heavy drinking^b^	61 (6.3%)	130 (6.3%)	48 (6.9%)	239 (6.4%)	0.99
Binge drinking^c^	179 (18.5%)	272 (13.0%)	54 (5.3%)	505 (13.6%)	< 0.01

Abbreviations: BMI, body mass index; COPD, chronic obstructive pulmonary disease.

^a^ Chronic conditions asked about in BRFSS include asthma, cardiovascular disease (angina or coronary heart disease, myocardial infarction/heart attack, stroke), COPD, and diabetes.

^b^ Heavy drinking defined as > 14 drinks/week for males; > 7 drinks/week for females, according to CDC definition [32].

^c^ Binge drinking defined as ≥ 5 drinks for males on one occasion; ≥ 4 drinks for females on one occasion, according to CDC definition [32].

### 3.2 Routine Health Care

**FIGURE 1:**
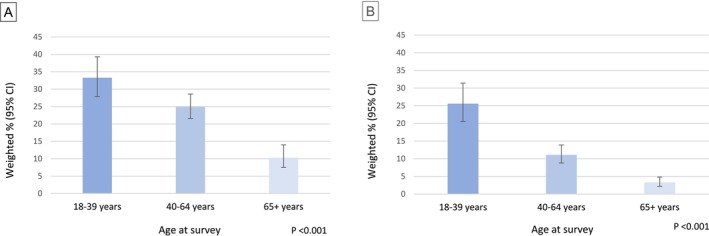
Prevalence and 95% confidence intervals (CI) of (A) no routine checkup within the past year and (B) not having a personal doctor, among CAYA survivors by age at survey.

Routine health care among CAYA survivors varied by age (Figure 1). More than one third (35.9%, 95% CI 30.3, 41.9) of young adults lacked routine care (> 1 year since routine checkup); the proportion lacking routine care was lower among middle age (25.6%, 95% CI 22.2, 29.3) and older adult CAYA survivors (11.3%, 95% CI 8.4, 15.0).

In the adjusted model (Table 3), young adults (aPR: 1.82; 95% CI 1.24, 2.67) or middle age (aPR 1.56; 95% CI 1.12, 2.17) CAYA survivors were more likely to lack routine health care. Additionally, being uninsured (aPR 1.90, 95% CI 1.51, 2.37) or unable to afford needed care (aPR 1.85, 95% CI 1.47, 2.34) was associated with lack of routine health care. CAYA survivors who were diagnosed as younger adolescents or young adults (ages 15–34) or those who were male were also more likely to lack routine health care. In analyses including primary cancer type (Table S1B), colorectal cancer was associated with lack of routine care, whereas those with endometrial cancers were more likely to receive routine care. Additionally, drinking any alcohol was associated with lack of routine health care in this model; other findings were similar to the full sample model.

**TABLE 3:** Factors associated with lack of routine care^a^ among CAYA survivors (*n* = 4212).

	aPR	95% CI	*p*
Age at cancer diagnosis, years			
0–14	1.32	(0.84, 2.06)	0.23
15–19	1.53	(1.07, 2.22)	0.02
20–24	1.52	(1.09, 2.12)	0.01
25–29	1.44	(1.06, 1.95)	0.02
30–34	1.37	(1.02, 1.85)	0.04
35–39	(Referent)		
Age at survey, years			
18–39	1.82	(1.24, 2.67)	< 0.01
40–64	1.63	(1.15, 2.31)	< 0.01
≥ 65	(Referent)		
Sex at birth			
Male	1.29	(1.01, 1.65)	0.04
Female	(Referent)		
Race and ethnicity			
NH^b^ API/AI/AN/Multiracial/Unk^c^	1.17	(0.80, 1.71)	0.41
NH Black	0.76	(0.42, 1.39)	0.38
Hispanic	0.83	(0.54, 1.29)	0.42
NH White	(Referent)		
Education			
High school or less	(Referent)		
Attended college	1.12	(0.89, 1.40)	0.34
Graduated college	1.03	(0.78, 1.37)	0.83
Marital status			
Divorced/separated/widowed	0.93	(0.74, 1.18)	0.57
Never married/unmarried couple	0.92	(0.68, 1.25)	0.60
Married	(Referent)		
Health insurance			
No	1.90	(1.51, 2.37)	< 0.01
Yes	(Referent)		
Annual income			
< $25,000	0.90	(0.65, 1.26)	0.58
$25,000–< $50,000	1.08	(0.81, 1.45)	0.58
≥ $50,000	(Referent)		
Smoking status			
Current	1.14	(0.89, 1.47)	0.31
Former	0.81	(0.62, 1.05)	0.11
Never	(Referent)		
Any alcohol (vs. no)	1.18	(0.96, 1.45)	0.11
Obese (vs. no)	0.87	(0.70, 1.07)	0.18
Number of chronic conditions			
0	(Referent)		
1	0.82	(0.64, 1.05)	0.11
≥ 2	0.89	(0.66, 1.19)	0.43
Number of cancers			
1	(Referent)		
2	0.75	(0.56, 1.00)	0.05
≥ 3	0.76	(0.40, 1.44)	0.40
Unable to afford care	1.85	(1.47, 2.34)	< 0.01
No survivorship care plan (vs. yes)^d^	1.24	(0.93, 1.65)	0.14

Abbreviations: aPR, adjusted prevalence ratio; CI, confidence interval.

^a^ Defined as > 1 year since last routine checkup.

^b^ Non‐Hispanic.

^c^ Includes Asian, Native Hawaiian/Pacific Islander, American Indian/Alaska Native, Multiracial, Unspecified Non‐Hispanic, Unknown, Not Sure, and Refused.

^d^ Missing included as a category.

### 3.3 Personal Doctor

One in four (25.6% 95% CI 20.5, 31.4) young adult CAYA survivors did not have a personal doctor (Figure 1). Similar to routine care, this proportion was lower among middle‐aged and older CAYA survivors.

Age at survey and insurance status were strongly associated with not having a personal doctor in adjusted models (Table 4). Specifically, young adults (aPR 3.14; 95% CI 1.84, 5.35) and middle‐aged CAYA survivors (aPR 2.16, 95% CI 1.38, 3.38) and those who were uninsured (aPR 3.23 95% CI: 2.40, 4.36) were significantly more likely to not have a personal doctor compared to older and insured CAYA survivors, respectively. Additionally, younger age at diagnosis, being male, having Hispanic ethnicity, or being divorced, separated, or widowed were associated with not having a personal doctor. In analyses including primary cancer type (Table S1C), cancer type was not associated with lacking a personal doctor. However, current smokers were more likely to lack a personal doctor, whereas those with 2 or more chronic conditions were more likely to have a personal doctor. Other findings were similar to the full sample model.

**TABLE 4:** Factors associated with not having a personal doctor among CAYA survivors (*n* = 4275).

	aPR	95% CI	*p*
Age at cancer diagnosis, years			
0–14	1.90	(1.03, 3.49)	0.04
15–19	2.49	(1.44, 4.32)	< 0.01
20–24	1.81	(1.01, 3.24)	0.05
25–29	1.64	(0.99, 2.71)	0.05
30–34	1.30	(0.78, 2.15)	0.31
35–39	(Referent)		
Age at survey, years			
18–39	3.14	(1.84, 5.35)	< 0.01
40–64	2.16	(1.38, 3.38)	< 0.01
≥ 65	(Referent)		
Sex at birth			
Male	1.78	(1.25, 2.53)	< 0.01
Female	(Referent)		
Race and ethnicity			
NH^a^ API/AI/AN/Multiracial/Unk^b^	1.32	(0.85, 2.07)	0.21
NH Black	1.20	(0.61, 2.40)	0.60
Hispanic	1.82	(1.21, 2.72)	< 0.01
NH White	(Referent)		
Education			
High school or less	(Referent)		
Attended college	1.00	(0.72, 1.40)	0.99
Graduated college	0.85	(0.54, 1.33)	0.49
Marital status			
Divorced/separated/widowed	1.44	(1.03, 2.00)	0.03
Never married/unmarried couple	1.28	(0.89, 1.85)	0.18
Married	(Referent)		
Health insurance			
No	3.23	(2.39, 4.36)	< 0.01
Yes	(Referent)		
Annual income			
< $25,000	0.72	(0.44, 1.17)	0.18
$25,000–< $50,000	0.78	(0.50, 1.23)	0.28
≥ $50,000	(Referent)		
Smoking status			
Current	1.37	(0.93, 2.01)	0.11
Former	1.09	(0.72, 1.64)	0.69
Never	(Referent)		
Any alcohol (vs. no)	1.04	(0.81, 1.40)	0.65
Obese (vs. no)	0.73	(0.53, 1.03)	0.07
Number of chronic conditions			
0	(Referent)		
1	1.03	(0.73, 1.49)	0.82
≥ 2	0.75	(0.45, 1.18)	0.20
Number of cancers			
1	(Referent)		
2	0.77	(0.49, 1.19)	0.23
≥ 3	0.51	(0.22, 1.18)	0.11
Unable to afford care	1.27	(0.96, 1.72)	0.12
No survivorship care plan (vs. yes)^c^	1.02	(0.70, 1.48)	0.73

Abbreviations: aPR, adjusted prevalence ratio; CI, confidence interval.

^a^ Non‐Hispanic.

^b^ Includes Asian, Native Hawaiian/Pacific Islander, American Indian/Alaska Native, Multiracial, Unspecified Non‐Hispanic, Unknown, Not Sure, and Refused.

^c^ Missing included as a category.

## 4 Discussion

In our study of a large, nationally representative sample of adult survivors of CAYA cancers, those who were young adults, middle age, or lacked insurance were more likely to be disconnected from routine health care and less likely to have a personal doctor, compared to older‐aged or insured CAYA survivors, respectively. Further, young adult CAYA survivors were more often uninsured and unable to afford needed care. Routine health care is critical to reducing modifiable risk factors and ensuring surveillance and early intervention for second cancers and chronic conditions. Our findings suggest that younger age in survivorship presents a critical window, as CAYA survivors are more likely to be disconnected from routine health care at a time when they might benefit most from necessary preventive services.

Comprehensive health care is essential across the lifespan for CAYA survivors [1, 16], given growing evidence of early onset of chronic conditions coupled with increased risk of second cancers and accelerated aging [15, 35, 36, 37]. While providing risk‐stratified survivorship care based on cancer‐ and treatment‐specific risks is important [38], the high prevalence of modifiable risk factors among young CAYA survivors in our study and others [39, 40, 41] highlights the importance of routine preventive care, including primary care. For example, over 40% of young adult CAYA survivors in our study had at least one chronic condition by age 39. Use of carcinogenic substances—including smoking and binge drinking—was common among young adult CAYA survivors, similar to other national studies [39, 40], and significantly higher than among older CAYA survivors. The hallmark of primary care is primary prevention to intervene on potential modifiable risks and screening for disease, including cancers [42, 43], and having a primary care provider (PCP) is associated with adherence to preventive health recommendations including cancer screenings [44].

Disconnection from routine health care among young adult CAYA survivors in our study suggests a need to strengthen pathways and support for health care transitions that occur as CAYA age. Developmental milestones in young adulthood may include moving, gaining/changing employment, pursuing education/training, developing romantic partnerships, and establishing a family [45]. Resulting changes in insurance, finances, and location may disrupt established health care relationships and access to care for younger CAYA survivors. Our findings regarding young adulthood as well as uninsurance and health care support the need for early, multilevel approaches to preventing and mitigating key barriers to comprehensive survivorship care. For example, a health insurance navigation intervention tool showed promise in improving health insurance literacy—including awareness of the Affordable Care Act—for adult survivors of childhood cancers [46]. Novel delivery methods like telehealth may also help engage and/or retain CAYA survivors in care during the highly mobile period of young adulthood [47]. Evaluations of telehealth to deliver cancer survivorship care have shown improved access, enhanced information exchange, and reduced barriers to access, including transportation and less time away from work [48, 49]. Further, telehealth interventions have improved quality of life for cancer survivors, reducing the treatment burden and disruption to survivors' lives. Survivors report that telehealth provides a personal experience, and that care from the home provides a familiar and relaxing environment [50], which may help reduce anxiety and avoidance rooted in medical trauma [51, 52]. Importantly, providers and survivors have reported telehealth would help improve the transition from pediatric to adult survivorship care [53].

In our study, CAYA survivors frequently reported an inability to afford needed care, and this was associated with a lack of routine health care, even after controlling for insurance status. Financial toxicity of treatment is common among CAYA survivors, and may be further compounded by disruptions to health and development that result in both high‐cost survivorship needs (e.g., fertility treatments, specialty care) and increased risk of unemployment, reliance on social security disability benefits, and lower incomes compared to the general population [26, 54, 55]. In 2017, a study comparing medical expenses of childhood cancer survivors to their siblings found that although both groups were insured, survivors were more likely to have high out‐of‐pocket expenses. They were also more likely to borrow money, not get prescriptions filled, and delay procedures [56]. Policies to reduce individual cost burden to CAYA survivors—who may experience compounding effects of the financial toxicity of treatment for many years—can reduce long‐term costs to the health system by facilitating prevention and early intervention through engagement in survivorship care.

### 4.1 Strengths and Limitations

Our study demonstrates that age in survivorship is an important factor in connection to care for CAYA survivors. A strength of our study includes the use of CDC data and a large, nationally representative sample of CAYA survivors, who are often treated in community and primary care settings [57]. Data were self‐reported, and cancer‐related questions were limited. Therefore, we lacked information on cancer stage/extent of disease, time since diagnosis, and type of cancer for some CAYA survivors. We were not able to assess urban or rural residence, given substantial missingness of available measures; future studies with robust residential information should explore geographic access and receipt of routine care among CAYA survivors. Our study was cross‐sectional, and population‐based studies with longitudinal measures of routine health care and more detailed cancer information are needed. Importantly, while adjusted models identified factors independently associated with outcomes, many of these factors are co‐occurring, particularly among the young adult CAYA survivor group.

## 5 Conclusions

Young adult CAYA survivors experience a triple threat of health risks: chronic conditions, modifiable risk factors, and disconnection from routine health care. National and local efforts to implement models of survivorship care and facilitate connection to care beyond provision of survivorship care plans should prioritize this population and must attend to disruptions in health insurance and financial toxicity of care. Such efforts are overdue [54, 55], and failure to act will have lifelong ramifications for this growing population of young survivors and the health systems that serve them.

## Author Contributions

**Andrea C. Betts:** conceptualization (lead), methodology (lead), writing – original draft (lead), writing – review and editing (lead). **Rebecca Eary:** conceptualization (lead), methodology (lead), writing – original draft (lead), writing – review and editing (equal). **Bhaskar Thakur:** formal analysis (lead), writing – original draft (supporting), writing – review and editing (supporting). **Amy Hughes:** writing – original draft (equal), writing – review and editing (equal). **Quiera S. Booker:** data curation (supporting), formal analysis (supporting), writing – original draft (supporting), writing – review and editing (supporting). **L. Aubree Shay:** writing – original draft (supporting), writing – review and editing (supporting). **Simon Craddock Lee:** writing – original draft (supporting), writing – review and editing (supporting). **Daniel C. Bowers:** writing – original draft (supporting), writing – review and editing (supporting). **Bijal A. Balasubramanian:** writing – original draft (equal), writing – review and editing (supporting).

## Disclosure

Andrea C. Betts reports consulting for Your Local Epidemiologist. This manuscript was prepared in part by Quiera S. Booker. The opinions expressed in this article are the author's own and do not reflect the views of the National Institutes of Health, the Department of Health and Human Services, or the United States government. All other authors have no disclosures.

## Conflicts of Interest

The authors declare no conflicts of interest.

## Supporting information


Table S1.

## Data Availability

The data underlying this study are available from the Behavioral Risk Factor Surveillance System, from the Centers for Disease Control and Prevention at https://www.cdc.gov/brfss/index.html.

## References

[1] S. H. Armenian and C. Chao , “Burden of Morbidity and Mortality in Adolescent and Young Adult Cancer Survivors,” Journal of Clinical Oncology 42, no. 6 (2024): 735–742, 10.1200/jco.23.01751. 37983585

[2] Institute NC , “Cancer in Children and Adolescents National Institutes of Health,” https://www.cancer.gov/types/childhood‐cancers/child‐adolescent‐cancers‐fact‐sheet#:~:text=As%20of%20January%201%2C%202020,alive%20in%20the%20United%20States.

[3] K. C. Oeffinger , A. C. Mertens , C. A. Sklar , et al., “Chronic Health Conditions in Adult Survivors of Childhood Cancer,” New England Journal of Medicine 355, no. 15 (2006): 1572–1582, 10.1056/NEJMsa060185. 17035650

[4] L. M. Turcotte , J. A. Whitton , D. L. Friedman , et al., “Risk of Subsequent Neoplasms During the Fifth and Sixth Decades of Life in the Childhood Cancer Survivor Study Cohort,” Journal of Clinical Oncology 33, no. 31 (2015): 3568–3575, 10.1200/jco.2015.60.9487. 26261260 PMC4622098

[5] R. Abrahão , R. C. Ribeiro , A. Brunson , and T. H. M. Keegan , “The Burden of Second Primary Cancers Among Childhood Cancer Survivors,” Annals of Cancer Epidemiology 4 (2020): 1–5.

[6] E. Suh , K. L. Stratton , W. M. Leisenring , et al., “Late Mortality and Chronic Health Conditions in Long‐Term Survivors of Early‐Adolescent and Young Adult Cancers: A Retrospective Cohort Analysis From the Childhood Cancer Survivor Study,” Lancet Oncology 21, no. 3 (2020): 421–435, 10.1016/s1470-2045(19)30800-9. 32066543 PMC7392388

[7] C. Ryder‐Burbidge , R. L. Diaz , R. D. Barr , et al., “The Burden of Late Effects and Related Risk Factors in Adolescent and Young Adult Cancer Survivors: A Scoping Review,” Cancers 13, no. 19 (2021): 4870, 10.3390/cancers13194870. 34638350 PMC8508204

[8] P. D. Inskip , A. J. Sigurdson , L. Veiga , et al., “Radiation‐Related New Primary Solid Cancers in the Childhood Cancer Survivor Study: Comparative Radiation Dose Response and Modification of Treatment Effects,” International Journal of Radiation Oncology, Biology, Physics 94, no. 4 (2016): 800–807, 10.1016/j.ijrobp.2015.11.046. 26972653 PMC5011040

[9] S. C. Adams , J. Herman , I. C. Lega , et al., “Young Adult Cancer Survivorship: Recommendations for Patient Follow‐Up, Exercise Therapy, and Research,” JNCI Journal of the National Cancer Institute 5, no. 1 (2021): pkaa099, 10.1093/jncics/pkaa099. PMC7919337 33681702

[10] L. B. Travis , W. Demark Wahnefried , J. M. Allan , M. E. Wood , and A. K. Ng , “Aetiology, Genetics and Prevention of Secondary Neoplasms in Adult Cancer Survivors,” Nature Reviews Clinical Oncology 10, no. 5 (2013): 289–301, 10.1038/nrclinonc.2013.41. 23529000

[11] M. M. Hudson , S. Bhatia , J. Casillas , and W. Landier , “Long‐Term Follow‐Up Care for Childhood, Adolescent, and Young Adult Cancer Survivors,” Pediatrics 148, no. 3 (2021): e2021053127, 10.1542/peds.2021-053127. 34462344 PMC9014377

[12] “Long‐Term Follow‐Up Guidelines for Survivors of Childhood, Adolescent and Young Adult Cancers, Version 5.0,” https://www.survivorshipguidelines.org. 10.1001/jamaoncol.2024.6812 PMC12188901 39976936

[13] A. W. Smith , K. M. Bellizzi , T. H. Keegan , et al., “Health‐Related Quality of Life of Adolescent and Young Adult Patients With Cancer in the United States: The Adolescent and Young Adult Health Outcomes and Patient Experience Study,” Journal of Clinical Oncology 31, no. 17 (2013): 2136–2145, 10.1200/jco.2012.47.3173. 23650427 PMC3731979

[14] M. E. McGrady , V. W. Willard , A. M. Williams , and T. M. Brinkman , “Psychological Outcomes in Adolescent and Young Adult Cancer Survivors,” Journal of Clinical Oncology 42, no. 6 (2024): 707–716, 10.1200/jco.23.01465. 37967297 PMC13019686

[15] N. Bhakta , Q. Liu , K. K. Ness , et al., “The Cumulative Burden of Surviving Childhood Cancer: An Initial Report From the St Jude Lifetime Cohort Study (SJLIFE),” Lancet 390, no. 10112 (2017): 2569–2582, 10.1016/s0140-6736(17)31610-0. 28890157 PMC5798235

[16] L. Nekhlyudov , M. A. Mollica , P. B. Jacobsen , D. K. Mayer , L. N. Shulman , and A. M. Geiger , “Developing a Quality of Cancer Survivorship Care Framework: Implications for Clinical Care, Research, and Policy,” Journal of the National Cancer Institute 111, no. 11 (2019): 1120–1130, 10.1093/jnci/djz089. 31095326 PMC6855988

[17] K. C. Oeffinger and M. S. McCabe , “Models for Delivering Survivorship Care,” Journal of Clinical Oncology 24, no. 32 (2006): 5117–5124, 10.1200/jco.2006.07.0474. 17093273

[18] P. C. Nathan , M. L. Greenberg , K. K. Ness , et al., “Medical Care in Long‐Term Survivors of Childhood Cancer: A Report From the Childhood Cancer Survivor Study,” Journal of Clinical Oncology 26, no. 27 (2008): 4401–4409, 10.1200/jco.2008.16.9607. 18802152 PMC2653112

[19] L. Moubadder , L. J. Collin , R. Nash , et al., “Drivers of Racial, Regional, and Socioeconomic Disparities in Late‐Stage Breast Cancer Mortality,” Cancer 128, no. 18 (2022): 3370–3382, 10.1002/cncr.34391. 35867419

[20] Statistics NCfH , “Health Insurance and Access to Care,” NCHS Fact Sheet: February 2017, https://www.cdc.gov/nchs/data/factsheets/factsheet_hiac.pdf.

[21] M. E. Price , N. Done , and S. D. Pizer , “The Relationship Between Follow‐Up Appointments and Access to Primary Care,” Journal of General Internal Medicine 35, no. 6 (2020): 1678–1683, 10.1007/s11606-020-05785-3. 32221854 PMC7280427

[22] L. L. Robison and M. M. Hudson , “Survivors of Childhood and Adolescent Cancer: Life‐Long Risks and Responsibilities,” Nature Reviews. Cancer 14, no. 1 (2014): 61–70, 10.1038/nrc3634. 24304873 PMC6425479

[23] K. A. Devine , A. Viola , P. Capucilli , O. J. Sahler , and J. R. Andolina , “Factors Associated With Noncompliance With Long‐Term Follow‐Up Care Among Pediatric Cancer Survivors,” Journal of Pediatric Hematology/Oncology 39, no. 3 (2017): 167–173, 10.1097/mph.0000000000000744. 28060114 PMC5364048

[24] R. Rehman , G. Solorzano , R. Heist , S. N. Thompson , and M. Badawi , “Predictors of Poor Adherence to Follow‐Up Care in Survivors of Childhood Cancer,” Oncology 36, no. 6 (2022): 350–354, 10.46883/2022.25920964. 35723943

[25] A. C. Kirchhoff , C. R. Lyles , M. Fluchel , J. Wright , and W. Leisenring , “Limitations in Health Care Access and Utilization Among Long‐Term Survivors of Adolescent and Young Adult Cancer,” Cancer 118, no. 23 (2012): 5964–5972, 10.1002/cncr.27537. 23007632

[26] M. A. Fiala , “Disparities in Health Care Affordability Among Childhood Cancer Survivors Persist Following the Affordable Care Act,” Pediatric Blood & Cancer 68, no. 12 (2021): e29370, 10.1002/pbc.29370. 34626446

[27] S. H. M. Janssen , W. T. A. van der Graaf , D. J. Meer , E. Manten‐Horst , and O. Husson , “Adolescent and Young Adult (AYA) Cancer Survivorship Practices: An Overview,” Cancers 13, no. 19 (2021): 4847, 10.3390/cancers13194847. 34638332 PMC8508173

[28] CDC (CfDCaP) , Behavioral Risk Factor Surveillance System Survey Data 2012, 2014, 2016–2019 (U.S. Department of Health and Human Services, Centers for Disease Control and Prevention, 2008).

[29] A. C. Betts , C. C. Murphy , L. A. Shay , B. A. Balasubramanian , C. Markham , and M. Allicock , “Polypharmacy and Prescription Medication Use in a Population‐Based Sample of Adolescent and Young Adult Cancer Survivors,” Journal of Cancer Survivorship 17, no. 4 (2022): 1149–1160, 10.1007/s11764-021-01161-0. 34997910 PMC10614319

[30] C. C. Murphy , H. M. Fullington , C. A. Alvarez , et al., “Polypharmacy and Patterns of Prescription Medication Use Among Cancer Survivors,” Cancer 124, no. 13 (2018): 2850–2857, 10.1002/cncr.31389. 29645083 PMC6147245

[31] Institute NC , “Adolescents and Young Adults With Cancer,” National Institutes of Health, https://www.cancer.gov/types/aya.

[32] Prevention CfDCa , “Alcohol and Public Health,” Division of Population Health, National Center for Chronic Disease Prevention and Health Promotion, Centers for Disease Control and Prevention, U.S. Department of Health and Human Services, https://www.cdc.gov/alcohol/index.htm.

[33] Stata Statistical Software: Release 18 (StataCorp LLC, 2023).

[34] L. A. Shay , H. M. Parsons , and S. W. Vernon , “Survivorship Care Planning and Unmet Information and Service Needs Among Adolescent and Young Adult Cancer Survivors,” Journal of Adolescent and Young Adult Oncology 6, no. 2 (2017): 327–332, 10.1089/jayao.2016.0053. 28103126 PMC5467130

[35] S. H. Armenian , C. J. Gibson , R. C. Rockne , and K. K. Ness , “Premature Aging in Young Cancer Survivors,” Journal of the National Cancer Institute 111, no. 3 (2019): 226–232, 10.1093/jnci/djy229. 30715446

[36] C. Chao , S. Bhatia , L. Xu , et al., “Chronic Comorbidities Among Survivors of Adolescent and Young Adult Cancer,” Journal of Clinical Oncology 38, no. 27 (2020): 3161–3174, 10.1200/jco.20.00722. 32673152 PMC7499612

[37] D. J. van der Meer , W. T. A. van der Graaf , D. van de Wal , H. E. Karim‐Kos , and O. Husson , “Long‐Term Second Primary Cancer Risk in Adolescent and Young Adult (15–39 Years) Cancer Survivors: A Population‐Based Study in The Netherlands Between 1989 and 2018,” ESMO Open 9, no. 1 (2024): 102203, 10.1016/j.esmoop.2023.102203. 38171190 PMC10837779

[38] C. Signorelli , J. E. Fardell , C. E. Wakefield , K. Webber , and R. J. Cohn , “The Cost of Cure: Chronic Conditions in Survivors of Child, Adolescent, and Young Adult Cancers,” in Cancer and Chronic Conditions: Addressing the Problem of Multimorbidity in Cancer Patients and Survivors, ed. B. Koczwara (Springer, 2016), 371–420.

[39] S. Kaul , S. P. Veeranki , A. M. Rodriguez , and Y. F. Kuo , “Cigarette Smoking, Comorbidity, and General Health Among Survivors of Adolescent and Young Adult Cancer,” Cancer 122, no. 18 (2016): 2895–2905. 27286172 10.1002/cncr.30086

[40] A. C. Betts , L. A. Shay , M. Allicock , S. M. Preston , A. Grimes , and C. C. Murphy , “Impact of the Early COVID‐19 Pandemic Among a National Sample of Adolescent and Young Adult Cancer Survivors,” Journal of Adolescent and Young Adult Oncology 12, no. 3 (2023): 324–330. 36173754 10.1089/jayao.2022.0045

[41] X. Ji , J. R. Cummings , A. C. Mertens , H. Wen , and K. E. Effinger , “Substance Use, Substance Use Disorders, and Treatment in Adolescent and Young Adult Cancer Survivors—Results From a National Survey,” Cancer 127, no. 17 (2021): 3223–3231, 10.1002/cncr.33634. 33974717

[42] G. Rubin , A. Berendsen , S. M. Crawford , et al., “The Expanding Role of Primary Care in Cancer Control,” Lancet Oncology 16, no. 12 (2015): 1231–1272, 10.1016/s1470-2045(15)00205-3. 26431866

[43] Office of Disease Prevention and Health Promotion OotASoH, Office of the Secretary, U.S. Department of Health and Human Services , “Healthy People 2030: Access to Primary Care,” https://health.gov/healthypeople/priority‐areas/social‐determinants‐health/literature‐summaries/access‐primary‐care.

[44] S. J. Atlas , R. W. Grant , T. G. Ferris , Y. Chang , and M. J. Barry , “Patient–Physician Connectedness and Quality of Primary Care,” Annals of Internal Medicine 150, no. 5 (2009): 325–335. 19258560 10.7326/0003-4819-150-5-200903030-00008 PMC2975389

[45] J. S. Mandelblatt , T. A. Ahles , M. E. Lippman , et al., “Applying a Life Course Biological Age Framework to Improving the Care of Individuals With Adult Cancers: Review and Research Recommendations,” JAMA Oncologia 7, no. 11 (2021): 1692–1699, 10.1001/jamaoncol.2021.1160. PMC8602673 34351358

[46] E. R. Park , A. C. Kirchhoff , K. A. Kuhlthau , et al., “A Health Insurance Navigation Intervention Tool (HINT) for Survivors of Childhood Cancer: Randomized Pilot Trial Results From the Childhood Cancer Survivor Study,” Journal of Clinical Oncology 40, no. 16_suppl (2022): 6508, 10.1200/JCO.2022.40.16_suppl.6508.

[47] A. J. Elder , H. Alazawi , F. Shafaq , A. Ayyad , and R. Hazin , “Teleoncology: Novel Approaches for Improving Cancer Care in North America,” Cureus 15, no. 8 (2023): e43562, 10.7759/cureus.43562. 37719501 PMC10502915

[48] R. J. Chan , M. Crichton , F. Crawford‐Williams , et al., “The Efficacy, Challenges, and Facilitators of Telemedicine in Post‐Treatment Cancer Survivorship Care: An Overview of Systematic Reviews,” Annals of Oncology 32, no. 12 (2021): 1552–1570, 10.1016/j.annonc.2021.09.001. 34509615

[49] L. B. Kenney , L. M. Vrooman , E. D. Lind , et al., “Virtual Visits as Long‐Term Follow‐Up Care for Childhood Cancer Survivors: Patient and Provider Satisfaction During the COVID‐19 Pandemic,” Pediatric Blood & Cancer 68, no. 6 (2021): e28927, 10.1002/pbc.28927. 33559385 PMC7995169

[50] A. Cox , G. Lucas , A. Marcu , et al., “Cancer Survivors' Experience With Telehealth: A Systematic Review and Thematic Synthesis,” Journal of Medical Internet Research 19, no. 1 (2017): e11, 10.2196/jmir.6575. 28069561 PMC5259589

[51] S. C. Vuotto , K. M. Perez , K. R. Krull , and T. M. Brinkman , “A Narrative Review of the Occurrence of Posttraumatic Stress Responses in Adolescent and Young Adult Cancer Survivors,” Clinical Oncology in Adolescents and Young Adults 5 (2015): 19–33, 10.2147/COAYA.S49175.

[52] B. Zebrack , M. Kwak , J. Salsman , et al., “The Relationship Between Posttraumatic Stress and Posttraumatic Growth Among Adolescent and Young Adult (AYA) Cancer Patients,” Psycho‐Oncology 24, no. 2 (2015): 162–168, 10.1002/pon.3585. 24916740 PMC4263687

[53] A. G. Costello , B. D. Nugent , N. Conover , A. Moore , K. Dempsey , and J. M. Tersak , “Shared Care of Childhood Cancer Survivors: A Telemedicine Feasibility Study,” Journal of Adolescent and Young Adult Oncology 6, no. 4 (2017): 535–541, 10.1089/jayao.2017.0013. 28657408 PMC5725630

[54] G. Di Giuseppe , L. Pagalan , A. Jetha , P. Pechlivanoglou , and J. D. Pole , “Financial Toxicity Among Adolescent and Young Adult Cancer Survivors: A Systematic Review of Educational Attainment, Employment, and Income,” Critical Reviews in Oncology/Hematology 183 (2023): 103914, 10.1016/j.critrevonc.2023.103914. 36706969

[55] S. Ruiz , M. M. Hudson , M. J. Ehrhardt , J. Maki , N. Ackermann , and E. A. Waters , “Childhood Cancer Survivors, Financial Toxicity, and the Need for Multilevel Interventions,” Pediatrics 152, no. 1 (2023): e2022059951, 10.1542/peds.2022-059951. 37264509

[56] R. D. Nipp , A. C. Kirchhoff , D. Fair , et al., “Financial Burden in Survivors of Childhood Cancer: A Report From the Childhood Cancer Survivor Study,” Journal of Clinical Oncology 35, no. 30 (2017): 3474–3481, 10.1200/jco.2016.71.7066. 28817372 PMC5648170

[57] L. C. Pinheiro , M. Rajan , M. M. Safford , D. M. Nanus , and L. M. Kern , “Nearly All Cancer Survivors Return to Primary Care,” Journal of the American Board of Family Medicine 35, no. 4 (2022): 827–832, 10.3122/jabfm.2022.04.220007. 35896447 PMC9365268

