# Supplementary material for: Lack of Routine Health Care Among Adult Survivors of Childhood, Adolescent, and Young Adult Cancers (CAYA): A Nationally Representative Study of 4300 Survivors
Source: Cancer Med. 2025 May 7;14(9):e70924. doi: 10.1002/cam4.70924 (PMC12056925; doi:10.1002/cam4.70924)
Supplement: Supplementary file 1 — Table S1. [file CAM4-14-e70924-s001.docx]

|  | N (wt %) |
| --- | --- |
| Brain | 38 (1.9%) |
| Breast | 342 (8.0%) |
| Cervical | 983 (30.9%) |
| Colorectal | 82 (2.7%) |
| Endometrial | 163 (4.1%) |
| Head and neck | 220 (7.0%) |
| Leukemia | 45 (1.4%) |
| Lymphoma | 133 (3.9%) |
| Lung | 24 (0.8%) |
| Melanoma | 528 (17.3%) |
| Ovarian | 146 (4.3%) |
| Testicular | 82 (2.3%) |
| Other | 364 (11.2%) |
| Unknown/not sure/refused | 142 (4.3%) |
| Missing | 3 (0.1%) |
|  |  |

Supplementary Table A. Cancer type at first diagnosis among CAYA survivors diagnosed with only one cancer (n=3,295)

Supplementary Table B. Factors associated with lack of routine care^a^ among CAYA survivors whose first cancer type is known (n=3,237)

|  | aPR | 95 % CI | | p | | | |
| --- | --- | --- | --- | --- | --- | --- | --- |
| Age at cancer diagnosis, years |  |  |  | | |  |  |
| 0-14 | 1.37 | (0.86, | 2.18) | | | 0.17 |  |
| 15-19 | 1.81 | (1.20, | 2.72) | | | <0.01 |  |
| 20-24 | 1.77 | (1.23, | 2.51) | | | <0.01 |  |
| 25-29 | 1.86 | (1.34, | 2.56) | | | <0.01 |  |
| 30-34 | 1.40 | (1.01, | 1.95) | | | 0.04 |  |
| 35-39 | (Referent) |  |  | | |  |  |
| Age at survey, years |  |  |  | | |  |  |
| 18-39 | 1.66 | (1.10, | 2.52) | | | 0.02 |  |
| 40-64 | 1.55 | (1.04, | 2.32) | | | 0.03 |  |
| ≥65 | (Referent) |  |  | | |  |  |
| Cancer type |  |  |  | | |  |  |
| Breast | 1.04 | (0.59, | 1.84) | | | 0.88 |  |
| Cervical | 0.80 | (0.52, | 1.25) | | | 0.33 |  |
| Endometrial | 0.37 | (0.14, | 0.96) | | | 0.04 |  |
| Ovarian | 1.09 | (0.59, | 2.05) | | | 0.77 |  |
| Head and heck | 0.88 | (0.52, | 1.48) | | | 0.63 |  |
| Colorectal | 2.02 | (1.11 | 3.67) | | | 0.02 |  |
| Lymphoma | (Referent) |  |  | | |  |  |
| Leukemia | 1.07 | (0.45, | 2.58) | | | 0.88 |  |
| Testicular | 0.58 | (0.30, | 1.12) | | | 0.11 |  |
| Melanoma | 0.99 | (0.63, | 1.57) | | | 0.98 |  |
| Lung | 0.93 | (0.36, | 2.38) | | | 0.88 |  |
| Brain | 1.11 | (0.45, | 2.76) | | | 0.82 |  |
| Other | 0.95 | (0.59, | 1.53) | | | 0.83 |  |
| Sex at birth |  |  |  | | |  |  |
| Male | 1.34 | (1.00, | 1.79) | | | 0.05 |  |
| Female | (Referent) |  |  | | |  |  |
| Race & ethnicity |  |  |  | | |  |  |
| NH^b^ API / AI / AN / Multiracial / Unk^c^ | 1.09 | (0.72, | 1.65) | | | 0.67 |  |
| NH Black | 0.73 | (0.45, | 1.19) | | | 0.20 |  |
| Hispanic | 1.01 | (0.70, | 1.46) | | | 0.95 |  |
| NH White | (Referent) |  |  | | |  |  |
| Cancer type |  |  |  | | |  |  |
| Breast | 1.04 | (0.59, | 1.84) | | | 0.88 |  |
| Cervical | 0.80 | (0.52, | 1.25) | | | 0.33 |  |
| Endometrial | 0.37 | (0.14, | 0.96) | | | 0.04 |  |
| Ovarian | 1.09 | (0.59, | 2.05) | | | 0.77 |  |
| Head and heck | 0.88 | (0.52, | 1.48) | | | 0.63 |  |
| Colorectal | 2.02 | (1.11 | 3.67) | | | 0.02 |  |
| Lymphoma | (Referent) |  |  | | |  |  |
| Leukemia | 1.07 | (0.45, | 2.58) | | | 0.88 |  |
| Testicular | 0.58 | (0.30, | 1.12) | | | 0.11 |  |
| Melanoma | 0.99 | (0.63, | 1.57) | | | 0.98 |  |
| Lung | 0.93 | (0.36, | 2.38) | | | 0.88 |  |
| Brain | 1.11 | (0.45, | 2.76) | | | 0.82 |  |
| Other | 0.95 | (0.59, | 1.53) | | | 0.83 |  |
| Education |  |  |  | | |  |  |
| High school or less | (Referent) |  |  | | |  |  |
| Attended college | 1.16 | (0.91, | 1.48) | | | 0.23 |  |
| Graduated college | 1.07 | (0.78, | 1.46) | | | 0.68 |  |
| Marital status |  |  |  | | |  |  |
| Divorced / Separated / Widowed | 0.94 | (0.73, | 1.21) | | | 0.62 |  |
| Never married / Unmarried couple | 0.86 | (0.63, | 1.17) | | | 0.34 |  |
| Married | (Referent) |  |  | | |  |  |
| Health insurance |  |  |  | | |  |  |
| No | 1.79 | (1.42, | 2.67) | | | <0.01 |  |
| Yes | (Referent) |  |  | | |  |  |
| Annual income |  |  |  | | |  |  |
| < $25,000 | 0.99 | (0.68, | 1.44) | | | 0.94 |  |
| $25,000 – < $50,000 | 1.16 | (0.85, | 1.60) | | | 0.35 |  |
| ≥$ 50,000 | (Referent) |  |  | | |  |  |
| Smoking status |  |  |  | | |  |  |
| Current | 1.24 | (0.96, | 1.61) | | | 0.10 |  |
| Former | 0.83 | (0.63, | 1.09) | | | 0.18 |  |
| Never | (Referent) |  |  | | |  |  |
| Any alcohol (vs. no) | 1.25 | (1.02, | 1.53) | | | 0.03 |  |
| Obese (vs. no) | 0.94 | (0.75, | 1.18) | | | 0.58 |  |
| Number of chronic conditions |  |  |  | | |  |  |
| 0 | (Referent) |  |  | | |  |  |
| 1 | 0.82 | (0.64, | 1.05) | | | 0.12 |  |
| ≥2 | 0.88 | (0.65, | 1.18) | | | 0.39 |  |
| Unable to afford care | 1.77 | (1.39, | 2.25) | | <0.01 | | |
| No survivorship care plan (vs. yes)^d^ | 1.23 | (0.94, | 1.63) | | 0.14 | | |

Abbreviations: aPR, adjusted prevalence ratio, CI, confidence interval
^a^ Defined as >1 year since last routine checkup

^b^ Non-Hispanic

^c^ Includes Asian, Native Hawaiian / Pacific Islander, American Indian / Alaska
Native, Multiracial, Unspecified Non-Hispanic, Unknown, Not Sure, and Refused

^d^ Missing included as a category

Supplementary Table C. Factors associated with not having a personal doctor among CAYA survivors whose first cancer type is known (n=3,285)

|  | aPR | | 95 % CI | | | | p | | |  |
| --- | --- | --- | --- | --- | --- | --- | --- | --- | --- | --- |
| Age at cancer diagnosis, years |  | |  | |  | |  | | |  |
| 0-14 | 2.05 | | (1.03, | | 4.08) | | 0.04 | | |  |
| 15-19 | 2.62 | | (1.42, | | 4.86) | | <0.01 | | |  |
| 20-24 | 1.88 | | (0.99, | | 3.57) | | 0.05 | | |  |
| 25-29 | 1.67 | | (0.95, | | 2.95) | | 0.08 | | |  |
| 30-34 | 1.21 | | (0.72, | | 2.06) | | 0.47 | | |  |
| 35-39 | (Referent) | |  | |  | |  | | |  |
| Age at survey, years |  | |  | |  | |  | | |  |
| 18-39 | 3.61 | | (2.02, | | 6.46) | | <0.01 | | |  |
| 40-64 | 2.70 | | (1.60, | | 4.56) | | <0.01 | | |  |
| ≥65 | (Referent) | |  | |  | |  | | |  |
| Sex at birth |  | |  | |  | |  | | |  |
| Male | 1.80 | | (1.15, | | 2.82) | | <0.01 | | |  |
| Female | (Referent) | |  | |  | |  | | |  |
| Race & ethnicity |  | |  | |  | |  | | |  |
| NH^a^ API / AI / AN / Multiracial /Unk^b^ | 1.37 | | (0.80, | | 2.35) | | 0.26 | | |  |
| NH Black | 1.15 | | (0.63, | | 2.07) | | 0.65 | | |  |
| Hispanic | 1.99 | | (1.28, | | 3.09) | | <0.01 | | |  |
| NH White | (Referent) | |  | |  | |  | | |  |
| Cancer type | |  | |  | |  | | |  | |
| Breast | 0.97 | | (0.43, | | 2.20) | | | 0.95 | |  |
| Cervical | 0.84 | | (0.43, | | 1.61) | | | 0.59 | |  |
| Endometrial | 0.79 | | (0.33, | | 1.88) | | | 0.59 | |  |
| Ovarian | 0.75 | | (0.31, | | 1.77) | | | 0.51 | |  |
| Head and heck | 0.90 | | (0.40, | | 2.05) | | | 0.80 | |  |
| Colorectal | 1.99 | | (0.75, | | 5.28) | | | 0.17 | |  |
| Lymphoma | (Referent) | |  | |  | | |  | |  |
| Leukemia | 0.92 | | (0.42, | | 2.03) | | | 0.84 | |  |
| Testicular | 0.74 | | (0.23, | | 2.39) | | | 0.62 | |  |
| Melanoma | 1.20 | | (0.65, | | 2.22) | | | 0.57 | |  |
| Lung | 1.17 | | (0.38, | | 3.62) | | | 0.79 | |  |
| Brain | 1.12 | | (0.39, | | 3.21) | | | 0.83 | |  |
| Other | 0.59 | | (0.27, | | 1.29) | | | 0.91 | |  |
| Education |  | |  | |  | |  | | |  |
| High school or less | (Referent) | |  | |  | |  | | |  |
| Attended college | 1.02 | | (0.71, | | 1.47) | | 0.90 | | |  |
| Graduated college | 0.93 | | (0.56, | | 1.53) | | 0.76 | | |  |
| Marital status |  | |  | |  | |  | | |  |
| Divorced / Separated / Widowed | 1.54 | | (1.05, | | 2.26) | | 0.03 | | |  |
| Never married / Unmarried couple | 1.35 | | (0.90, | | 2.03) | | 0.27 | | |  |
| Married | (Referent) | |  | |  | |  | | |  |
| Health insurance |  | |  | |  | |  | | |  |
| No | 3.23 | | (2.34, | | 4.48) | | <0.01 | | |  |
| Yes | (Referent) | |  | |  | |  | | |  |
| Annual income |  | |  | |  | |  | | |  |
| < $25,000 | 0.70 | | (0.41, | | 1.21) | | 0.20 | | |  |
| $25,000 – < $50,000 | 0.79 | | (0.47, | | 1.33) | | 0.38 | | |  |
| ≥$ 50,000 | (Referent) | |  | |  | |  | | |  |
| Smoking status |  | |  | |  | |  | | |  |
| Current | 1.58 | | (1.07, | | 2.32) | | 0.02 | | |  |
| Former | 1.09 | | (0.68, | | 1.73) | | 0.73 | | |  |
| Never | (Referent) | |  | |  | |  | | |  |
| Any alcohol (vs. no) | 1.10 | | (0.83, | | 1.46) | | 0.51 | | |  |
| Obese (vs. no) | 0.75 | | (0.52, | | 1.10) | | 0.14 | | |  |
| Number of chronic conditions |  | |  | |  | |  | | |  |
| 0 | (Referent) | |  | |  | |  | | |  |
| 1 | 1.05 | | (0.72, | | 1.52) | | 0.80 | | |  |
| ≥2 | 0.57 | | (0.36, | | 0.93) | | 0.02 | | |  |
| Unable to afford care | 1.34 | | (0.98, | | 1.83) | | 0.07 | | |  |
| No survivorship care plan (vs. yes)^b^ | 0.94 | | (0.65, | | 1.37) | | 0.76 | | |  |

Abbreviations: aPR, adjusted prevalence ratio; CI confidence interval
^a^ Non-Hispanic ^b^ Includes Asian, Native Hawaiian / Pacific Islander, American Indian / Alaska
Native, Multiracial, Unspecified Non-Hispanic, Unknown, Not Sure, and Refused
^b^ Missing included as a category
